# ATORVASTATIN CAN PREVENT HEPATIC REMOTE REPERFUSION INJURY

**DOI:** 10.1590/0102-6720201700030008

**Published:** 2017

**Authors:** Carlos Henrique Marques dos SANTOS, Doroty Mesquita DOURADO, Baldomero Antonio Kato da SILVA, Henrique Budib Dorsa PONTES, Euler de AZEVEDO-NETO, Giovanna Serra da Cruz VENDAS, Ian de Oliveira CHAVES, João Victor Cunha MIRANDA

**Affiliations:** 1 Medicine Course, University Anhanguera-Uniderp, Campo Grande, MS,; 2 Physiotherapy Course, Federal Universityof Piauí, Parnaíba, PI, Brazil

**Keywords:** Ischemia, Reperfusion injury, Ischemic postconditioning, Hydroxymethylglutaryl-CoA reductase inhibitors, Liver

## Abstract

**Background:**

Some studies have shown that statins have a promising effect on protection against reperfusion injury.

**Aim:**

To evaluate the ability of ischemic postconditioning, statins and both associated to prevent or minimize reperfusion injury in the liver of rats subjected to ischemia and reperfusion by abdominal aorta clamping.

**Method:**

Were used 41 Wistar rats, which were distributed into five groups: ischemia and reperfusion (I/R), ischemic postcondictioning (IPC), postconditioning + statin (IPC+S), statin (S) and Sham. It was performed a medium laparotomy, dissection and isolation of the infra-renal abdominal aorta; excepting Sham group, all the others were submitted to the aorta clamping for 70 min (ischemia) and posterior clamping removing (reperfusion, 70 min). In the IPC and IPC+S groups, postconditioning was performed between the ischemia and reperfusion phases by four cycles of reperfusion and ischemia lasting 30 s each. In IPC+S and S groups, preceding the surgical procedure, administration of 3.4 mg/day of atorvastatin was performed for seven days by gavage. The left hepatic lobe was removed for histological study and euthanasia was performed.

**Results:**

The mean hepatic injury was 3 in the I/R group, 1.5 in the IPC group, 1.2 in the IPC+S group, 1.2 in the S group, and 0 in the SHAM group. The I/R group had a higher degree of tissue injury compared to the others in the statistical analysis and there was no difference between the others (p<0.01).

**Conclusion:**

Ischemic postconditioning and atorvastatin were able to minimize hepatic reperfusion injury, either alone or in combination.

## RESUMO

**Racional:**

Alguns estudos têm demonstrado que as estatinas apresentam efeito promissor contra a lesão de reperfusão.

**Objetivo:**

Avaliar a capacidade do pós-condicionamento, estatina e ambos associados em prevenir ou minimizar a lesão de reperfusão à distância no fígado em ratos submetidos à isquemia e reperfusão por clampeamento aórtico.

**Método:**

Foram utilizados 41 ratos Wistar distribuídos em cinco grupos: isquemia e reperfusão (I/R), pós-condicionamento isquêmico (PCI), pós-condicionamento + estatina (PCI+E), estatina (E) e SHAM. Os animais foram anestesiados, submetidos à laparotomia, dissecção e isolamento da aorta abdominal infrarrenal; exceto o grupo SHAM, todos os outros foram submetidos ao clampeamento aórtico por 70 min (isquemia) e posterior retirada do clampe (reperfusão). Nos grupos PCI e PCI+E o pós-condicionamento foi realizado entre as fases de isquemia e reperfusão por quatro ciclos de reperfusão e isquemia durando 30 s cada. Nos grupos PCI+E e E, previamente ao procedimento cirúrgico foi realizada a administração de 3,4 mg/dia de atorvastatina durante sete dias por gavagem.

**Resultados:**

A média de lesão hepática foi 3 no grupo I/R, 1,5 no grupo PCI, 1,2 no grupo PCI+E, 1,2 no grupo E e 0 no grupo SHAM. O grupo I/R teve maior grau de lesão tecidual (p<0,01).

**Conclusão:**

O pós-condicionamento isquêmico e atorvastatina foram capazes de minimizar a lesão hepática de reperfusão remota, isoladamente e em associação.

## INTRODUCTION

Reperfusion is a fundamental step in the treatment of ischemia. However, clinical and experimental evidence shows that the main events leading to cell and tissue dysfunction are related to reperfusion^7^ ^,^ ^22^.

The liver receives all blood from the splanchnic system, so that an aortic ischemia or its main branches will certainly lead to the arrival of the reactive oxygen species (ROS) to this organ and may cause intense hepatic injury at a distance, what may lead a multiple organ dysfunction^2^.

Aiming to address the various situations of ischemia avoiding reperfusion lesions, a large number of substances and procedures have been studied, including its remote and local effects. Some of the published proposals obtained good experimental results, but without proven success in clinical practice^12^ ^,^ ^13^.

In 2003, Zhao *et al*.^23^ proposed an alternative treatment of ischemia and reperfusion (IR), ischemic postconditioning (IPC), which consists of performing one or more cycles of reperfusion followed by one or more cycles of ischemia, before the reperfusion phase, demonstrating a protective effect on myocardial ischemia in animals.

In 2012, Onody*et al*.^15^found that IPC was able to prevent hepatic reperfusion injury at a distance, performing mesenteric IR, but in 2016, Santos *et al*.^17^have not shown the same results and there is therefore a question to be clarified as to the actual ability of the IPC to prevent such liver lesions.

Much has been studied about the pathophysiology of reperfusion injury and some mechanisms have already been well evidenced such as the role of free radicals, vascular endothelial dysfunction, and neutrophil-mediated injury^1^. Recently, there has been an increase in interest in statins, drugs known for their antidislipidemic effect, this time due to its pleiotropic effect, which is characterized by anti-inflammatory properties, immunomodulating, antithrombogenic actions and improvement of endothelial function^20^. Recent experimental studies^1^ have shown promising results with the use of statins demonstrating their role in the protection against IR injury, a fact that led us to inquire about their benefits facing reperfusion injury.

Thus, the aim of this study was to evaluate the capacity of IPC associated with the use of statins in reducing the tissue injury of the liver.

## METHODS

The study was approved by the Committee of Ethics in Animal Experimentation of the University Anhanguera-Uniderp. A total of 41 Wistar norvergic male rats weighing 250-300g were collected from the Anhanguera-Uniderp University Animal Hospital. The animals were kept in cages at ambient temperature of approximately 23°C with 12h light cycles and received water and feed ad libitum.The animals were distributed in the following groups: 1) ischemia and reperfusion group (I/R): nine rats were submitted to ischemia for 70 min by aortic clamping, followed by reperfusion of 70 min; 2) ischemic postconditioning group (IPC): nine rats were submitted to the ischemia procedure for 70 min by aortic clamping and reperfusion for 70 min and between ischemia and reperfusion, four cycles of reperfusion (30 s each) were performed, interspersed by four cycles of ischemia (30 s each); 3) ischemic postconditioning + statin group (IPC+S): nine rats received 3.4mg/day of atorvastatin, one dose per day through the gavage method, for seven days and then were submitted to the ischemia procedure for 70 min by aortic clamping and reperfusion for 70 min, and between ischemia and reperfusion, four cycles of reperfusion (30 s each) were performed, interspersed by four cycles of ischemia (30 s each); 4) statin group (S): nine rats received 3.4mg/day of atorvastatin, one dose per day through the gavage method, for seven days, and then were subjected to the ischemia procedure for 70 min by aortic clamping and reperfusion for 70 min; 5) SHAM group: five rats submitted to laparotomy, dissection and isolation of infra-renal aorta.

The animals were anesthetized by intraperitoneal injection of a 2:1 solution of Cetamine Hydrochloride (Cetamin®), 50mg/ml, and Xylasine Hydrochloride (Xilazin®), 20mg/ml, respectively, at a dose of 0.1ml/100g).

After anesthesia, the rats were submitted to median longitudinal laparotomy of approximately 4 cm, exteriorization of the small intestine, identification and dissection of infra-renal abdominal aorta artery.

In all groups, except SHAM, the abdominal aorta was occluded by atraumatic vascular clamp that remained for 70 min (ischemia phase). After clamp placement, the small intestine was repositioned into the abdominal cavity and the surgical wound was closed with continuous suture of the skin with 4-0 monofilament nylon. After the ischemia phase, the abdominal wall was reopened by removal of the suture and in the I/R and S groups the vascular clamp was removed, initiating the reperfusion phase, lasting 70 min. In the IPC and IPC+S groups, preceding the reperfusion phase, the ischemic postconditioning was performed by four cycles of reperfusion (removal of the atraumatic vascular clamping of the abdominal aorta) with duration of 30 s each, interspersed by four cycles of ischemia (occlusion of the abdominal aorta artery by atraumatic vascular clamp), also with duration of 30 s each. 

In all groups after the beginning of the reperfusion phase, the abdomen was again closed by continuous suturing of the skin with 4-0 monofilament nylon thread until the end of the experiment.

In the SHAM group, only a median longitudinal laparotomy of approximately 4 cm was performed, exteriorization of the small intestine, identification and dissection of infra-renal abdominal aorta artery.

After the reperfusion phase, all animals were submitted to resection of the left hepatic lobe, and these specimens were washed with saline solution and placed in 10% formaldehyde solution for histological analysis.

Euthanasia was performed by intraperitoneal administration of a lethal dose of cetamine + xylazine hydrochloride (0.4ml/100g).

Slides were prepared with the harvested material, which were stained with hematoxylin-eosin and analyzed by optical microscopy by a single observer, without prior knowledge of it on the group belonging to each rat.

Liver segments were classified according to the degree of tissue injury according to Rhoden et al.^16^, taking into account the finding of vascular congestion (sinusoidal, centrolobular and portal space), necrosis and hepatic steatosis in: 0: absence of alterations; 1: changes in light intensity (less than 25% of the field analyzed); 2: changes of moderate intensity (25-50% of the field analyzed); 3: changes of severe intensity (more than 50% of the analyzed field).

### Statistical analysis

After the analysis of the data, the results were submitted to statistical treatment, using the Kruskal-Wallis non-parametric test, considering p<0.05.

## RESULTS

**FIGURE 1:**
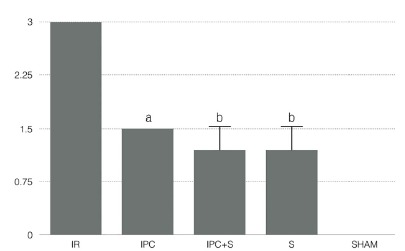
Comparison of the medians of the degrees of hepatic injury among the different groups analyzed

The averages of degrees of tissue injury were 3 in the I/R group, 1.5 in the IPC group, 1.2 in the IPC+S and S groups, and 0 in the SHAM group (Table 1 and Figure 1).

**TABLE 1:** Degree of histopathological lesion in the liver parenchyma in rats per group

Rats	Groups				
	I/R	IPC	IPC+S	S	SHAM
1	3	2	1	1	0
2	3	1	1	1	0
3	3	1	2	2	0
4	3	2	2	1	0
5	3	2	1	1	0
6	3	1	1	2	-
7	3	2	1	1	-
8	3	1	1	1	-
9	3	2	1	1	-
Average	3	1.5	1.2	1.2	0

Kruskal-Wallis; p<0,0001; “a” p<0.05 in relation to group IR; “b” p<0.01 in relation to group IR

**FIGURE 2:**
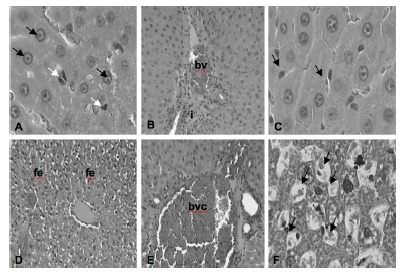
Photomicrographs of the main histological findings of the different groups according to the classification of Rhoden et al.: A) normal; white arrows: Kupfer cells; black arrows: hepatocytes; B) grade 1; bv= blood vessel; i= inflammation; C) degree 2; arrows: mild inflammatory infiltrate; D) grade 2; fe= focal edema; E) grade 2; bvc= blood vessel congestion; F) grade 3; arrows: tissue necrosis (H&E, 10x and 40x)

The main histological findings in the different groups can be observed in the Figure 2.

## DISCUSSION

Ischemia followed by reperfusion may induce apoptosis and an inflammatory response that affects tissue repair, especially in the lung. As a result, many have evaluated the impact of IPC on subsequent apoptotic and inflammatory responses. In experimental IR models in rats with 30 min of ischemia and 3 h of reperfusion there was a significant decrease in tissue necrosis with IPC. There is also a decrease in ROS generation and protection of mitochondrial integrity, suggesting that the protective effect of IPC may be the result of a reduction in the inflammatory response. However, few studies have directly assessed the impact of IPC on inflammation. IPC may limit the expression of P-selectin, which is required for neutrophil bearing and its recruitment. In addition, it may reduce the accumulation of neutrophils in the affected region, decrease adhesion to ischemic vascular endothelium, and attenuate the endothelial dysfunction of the involved vessel, events that normally occur in IR^8^.

In the present study, it was observed protection of the liver with IPC was demonstrated, demonstrating the efficacy of the method against this IR model, which may be justified by the fact that ROS, regardless of where they are produced, when reperfusion occurs, are spread throughout the body causing the remote reperfusion injury, so much that in the I/R group it was observed a marked injury in the liver. Acting as a moderator of ROS production, IPC can mitigate local and remote injury. Onody et al.^15^ demonstrated the same efficacy with IPC in remote protection of the liver by performing intestinal IR. However, as a method of evaluation, these authors used the dosage of transaminases, different from the one used here. Santos et al.^17^ also performed histological analysis, not confirming the efficacy of the method. The difference between the good result observed here and that published by Santos et al.^17^ may be due to the fact that these authors performed intestinal IR, whereas here aortic clamping was used. Also the RI periods were different between this study and that of Santos et al.^17^ (30 and 60 min IR, respectively). Perhaps the ischemia produced directly in the intestine, whose blood is fully drained to the liver leads to ROS overload, whereas in aortic IR this can be slowed down.

There are no studies with a similar design to that used here that allow direct comparison; But Seifi et al.^19^ also verified renal hepatic protection with renal IR in rats, as well as Costa et al.^3^ who applied IR to the hind limb of rats also observing protection with IPC in the liver of the animals. Thus, although there are few publications, there is good evidence of the efficacy of IPC in remote liver protection. However, theoretically there would be a greater advantage in a non-interventional method that would deliver as good or better results than IPC as a known and safe drug. This leads to great interest in the study of statins for this purpose.

In the present study, protection of the liver was obtained with the use of atorvastatin, at the same intensity as with IPC. As there are no studies with the same design used here, aortic clamping and use of atorvastatin, the comparison with the literature is also impaired. In addition, since the use of statins for the prevention of reperfusion injury is relatively new, the best route of administration and the optimal dose are items to be clarified in future research. Gastric tube administration was chosen here with the intention of simulating what is practiced in humans, that is, absorption by the gastrointestinal tract, aiming at its clinical application.

Statins were successfully tested for this purpose in several situations. Wu et al.^21^ performed renal IR in rats and demonstrated that atorvastatin decreased tissue damage in the control group. The same results were obtained by Cusomano et al.^4^ in the renal IR of rats using atorvastatin. Kocak et al.^11^ also confirmed the efficacy of simvastatin in hepatic protection in rats, but these authors applied IR directly to the hepatic hilum, thus not configuring a study of their effect at a distance, as presented here. These authors also applied the statin by single intraperitoneal injection, whereas in the present research gavage was used during one week. Considering the good initial results of statins under IR conditions in animals and the safety of these drugs, Sarim et al.^18^ evaluated their efficacy in humans in a study in which patients undergoing severe liver resections had previously used atorvastatin for at least three days. Postoperative transaminase levels were lower in these patients than in the control group. Again, it should be noted that it was not a remote-effect study, since IR was produced directly on the hepatic hilum.

Statins also protect other tissues in the presence of IR, such as heart^6^ ^,^ ^9^ ^,^ ^10^, nervous system^5^ and lung^14^. The protection mechanism of statin against situations of IR is due to its pleiotropic effect. Inhibiting the conversion of HMG-CoA to L-mevalonate, statins prevent the synthesis of isoprenoids, which are precursors of cholesterol biosynthesis, which serve as important lipid ligands for post-transductional modification of intracellular proteins such as GTPases, Rho, Rac, and Ras. This protein isoprenylation allows adequate subcellular localization and intracellular trafficking of proteins, which control various cellular functions, and the inhibition of these pathways may determine important components of the pleiotropic effects of statins. The Rho pathway is related to oxidative stress, atherosclerosis and high blood pressure, signaling the path between the two crucial mechanisms, such as cytoskeletal remodeling and ROS synthesis^11^.

In the development of this project it was not known that the therapeutic methods applied would find the results presented here, so that an association group (IPC+S) was created with the aim of improving tissue protection. However, there was no advantage in the association, since in isolation these therapeutic methods obtained mean tissue lesion statistically similar to the PCI and E groups. Thus, it is verified that atorvastatin has the capacity to protect the liver in remote reperfusion situations, in the same intensity of the IPC, and it is possible to invest in research that confirms the best method of using these therapies to apply them in clinical practice.

## CONCLUSION

Ischemic postconditioning and atorvastatin were able to minimize hepatic reperfusion injury, either alone or in combination.
